# Neurofilament light chain reference values in serum and cerebrospinal fluid: a bi-compartmental analysis in neurological diseases

**DOI:** 10.1007/s00415-025-13271-1

**Published:** 2025-07-25

**Authors:** Steffen Halbgebauer, Veronika Klose, Badrieh Fazeli, Paula Klassen, Christoforos Alexudis, Gabriele Nagel, Angela Rosenbohm, Dietrich Rothenbacher, Nerea Gomez de San Jose, Simon Witzel, Zeynep Elmas, Maximilian Wiesenfarth, Joachim Schuster, Johannes Dorst, Andre Huss, Franziska Bachhuber, Markus Otto, G. Bernhard Landwehrmeyer, Albert C. Ludolph, Hayrettin Tumani

**Affiliations:** 1 Department of Neurology, University Hospital Ulm, Oberer Eselsberg 45, 89081 Ulm, Germany; 2 German Center for Neurodegenerative Diseases (DZNE E.V.), Ulm, Germany; 3 Department of Neurology, University Hospital Halle, Halle (Saale), Germany; 4 Department of Neurology, Kempten Hospital, Kempten, Germany; 5 Institute for Epidemiology and Medical Biometry, Ulm University, Ulm, Germany

**Keywords:** Neurofilament, NfL, Age reference values, Z-score, Serum, CSF, Bi-compartment model

## Abstract

**Background:**

Concentrations of neurofilament light chain (NfL), a neuroaxonal damage marker, increase with age. Therefore, age-dependent reference values are important in clinical practice. However, so far these have only been established with a bead-based system and age-dependent z-scores for CSF are missing. In addition, we here propose how the combined analysis of CSF and serum NfL could help in the discrimination between central (CNS) and peripheral nervous system (PNS) axonal degeneration.

**Methods:**

For the calculation of age reference values, serum and CSF NfL concentrations from 1,514 control subjects, measured using the microfluidic Ella system, were applied.

**Results:**

Age-dependent NfL levels were calculated with additive quantile regression and presented with percentiles and z-scores. We observed a non-linear increase of NfL in serum and CSF. The spearman r of the association with age was 0.81 (95% CI 0.78–0.83), *p* < 0.0001 and 0.82 (95% CI 0.79–0.85), *p* < 0.0001 for serum and CSF NfL, respectively. Serum and CSF NfL levels were also associated with each other (*r* = 0.68 (95%CI 0.62–0.73), *p* < 0.0001). Furthermore, we used this association to establish a bi-compartmental CSF and serum NfL model allowing to differentiate between peripheral or central origin of neurodegeneration.

**Conclusions:**

The age reference curves corroborate findings of an exponential elevation of NfL in serum and CSF with increasing age. As NfL values from different platforms are not interchangeable, this is of additional high relevance. Moreover, the association between CSF and serum NfL values could be applied for clinical use regarding overlapping symptoms of CNS and PNS-based neurological diseases.

**Supplementary Information:**

The online version contains supplementary material available at 10.1007/s00415-025-13271-1.

## Background

Neurofilaments represent the principal cytoskeletal component of neurons, providing structural stability through the formation of fibrillary networks. They are highly abundant in neuronal axons, where they are involved in a number of tasks, including axonal transport and dendritic branching [31, 32]. The most abundant neurofilament is the neurofilament light chain (NfL), a 68 kDa intermediate filament protein. NfL is also the neurofilament that has been the subject of the most extensive investigation to date, serving as a fluid biomarker in neurological diseases [10]. Following neuroaxonal damage in the central nervous system (CNS) NfL is released into the CSF and subsequently drained into the bloodstream, where it can be quantified using state-of-the-art technology such as microfluidic assays (Ella) or single molecule array (Simoa). Given its expression pattern, NfL levels are markedly elevated in motor neuron diseases such as amyotrophic lateral sclerosis (ALS) [12, 23, 28], but increased levels can also be observed in numerous other conditions, including multiple sclerosis [4, 6] and frontotemporal dementia [13, 27]. This offers the opportunity to utilize NfL levels in CSF and blood as diagnostic, progression, and monitoring markers in the clinic and clinical trials. However, it should be noted that NfL levels can be affected by comorbidities (e.g., cardiovascular diseases, diabetes mellitus), confounding factors such as BMI or the glomerular filtration rate and, in particular, by age [8, 14]. A number of studies have been conducted to establish age-related reference NfL levels in control patients with a wide age range [3, 22, 29]. These studies employed different versions of the Simoa NfL kit, although other platforms have also been used in studies on NfL. One such platform is the microfluidic Ella NfL test, which has been shown to correlate well with the Simoa assays [11, 19, 25]. The Ella assay is applicable in daily clinical routine and utilizes the same antibodies as the Simoa kits. However, Simoa and Ella NfL kits demonstrate disparate absolute NfL concentrations. Consequently, the objective of this study was to establish age-related NfL reference values in serum and CSF using the microfluidic Ella NfL technology in subjects without neurodegenerative and neuroinflammatory diseases. Furthermore, we set up z-score references for serum and CSF NfL levels which can be applied across platforms. Our study covers a wide age range from 18 to 89 years (50.3% female) using in total 1514 NfL values for the age reference NfL concentration calculations. In addition, from approximately 400 control patients CSF and serum were available which we used to generate a bi-compartmental model to help distinguish between peripheral and central origins of neuroaxonal damage. To validate the bi-compartmental model we additionally analyzed CSF and serum NfL levels in four neurological disorders.

## Methods

### Patients

In this study, we used NfL values from control subjects from two different studies performed at Ulm University Hospital. The first cohort consisted of patients ranging from age 33 to 89 enrolled in the population-based ALS Swabian registry (ethics votes No. 11/10, No. B-F-2010-062 and No. 7/11300) as control cases (*n* = 576). The study has been previously described [18, 20, 26, 30]. All members of the registry are listed in the supplementary materials. From this cohort only serum NfL was available.

The second cohort comprised 72 patients with serum and CSF NfL concentrations available, ranging from age 21 to 88, which were used as control patients in a neurofilament NfL and NfH validation study [12]. To increase the number of samples we additionally analyzed serum NfL in 385 and CSF NfL in another 403 patients from age 18 to 89, which were seen at the Department of Neurology at Ulm University Hospital between 2014 and 2023 and collected through convenience sampling. These patients did not show clinical or radiological signs for neurodegeneration and an acute inflammation of the central nervous system was ruled out by CSF analysis (diagnoses are shown in Table S1). A flow chart of the patient selection is shown in the supplementary materials (Fig. S1).

For the evaluation of the bi-compartmental model 384 control patients for whom serum and CSF were available were analyzed for NfL. Additionally, we measured NfL from patients with a neurological disorder seen at the University Hospital Ulm (Fig. S1). The multiple sclerosis (MS) patients were diagnosed with progressive MS according to the revised McDonald criteria [24]. For the diagnosis of Guillain–Barré syndrome (GBS) the consensus guidelines according to Leonhard et al. 2019 [15] and for idiopathic intracranial hypertension (IIH) according to Mollan et al. 2018 [17] were applied. CSF and serum NfL levels of 10 MS, 9 GBS, and 12 IIH patients were analyzed for this study. CSF and serum NfL concentrations of 10 amyotrophic lateral sclerosis (ALS) patients were taken from a previously published study where ALS was diagnosed according the El Escorial criteria [5, 12]. Control and disease patients seen at the neurological department at the University Hospital Ulm suffering from acute or chronic renal insufficiency were excluded from this study. The examination was approved by the local ethics committee (approval number Ulm 20/10) and conducted following the Declaration of Helsinki. All participants gave their written informed consent to participate in the study.

### Sampling and NfL measurement

CSF and blood samples were collected by lumbar puncture and venous sampling, respectively. Samples were centrifuged at 2000 g for 10 min and CSF supernatant and the extracted serum aliquoted and frozen within 30 min at – 80 °C. Both CSF and serum samples were stored in polypropylene tubes.

For NfL quantification in CSF and serum the microfluidic Ella platform (Bio-Techne, Minneapolis, USA) was used. The analyses were performed according to the manufacturer’s instructions.

### Statistics

For the analysis of the effect of age on NfL levels due to a non-linear relation an additive quantile regression analysis was performed based on the control population using RStudio (Version 4.3.1). For visualization Python (Version 3.11.5) was applied. According to this analysis we calculated the z-sores for the control group. Moreover, we applied the outcome of the model to determine the z-scores of the disease cohorts.

GraphPad Prism V.10.3.1 (GraphPad, Software, La Jolla, California, USA) was used to calculate the association between serum and CSF NfL applying the spearman rank correlation and for the comparison between the diagnostic groups using Kruskal–Wallis followed by Dunn’s post hoc test. For female and male NfL level differences between age groups the Mann–Whitney *U* test was applied. A *p* < 0.05 was indicative of statistically significant results.

## Results

### NfL reference concentrations by age

A total of 1514 NfL concentrations were utilized for the establishment of reference values by age. The median NfL concentrations for the serum and CSF control cohort, along with the demographic parameters, are given in Table 1.

**Table 1:** Demographic and NfL parameters of the analyzed cohort

	Control patients for serum NfL	Control patients for CSF NfL	Subcohort with paired samples for CSF and serum
*N*	1033	475	384
Age (years) mean ± SD	56 ± 18	45 ± 19	43 ± 18
f/m	520/513	302/173	237/147
Serum NfL [pg/ml] Median (IQR)	19 (10–27)	n/a	9 (6–15)
CSF NfL [pg/ml] Median (IQR)	n/a	359 (218–589)	346 (215–544)

*CSF* cerebrospinal fluid, *f* female, *m* male, *NfL* neurofilament light chain

**Fig. 1:**
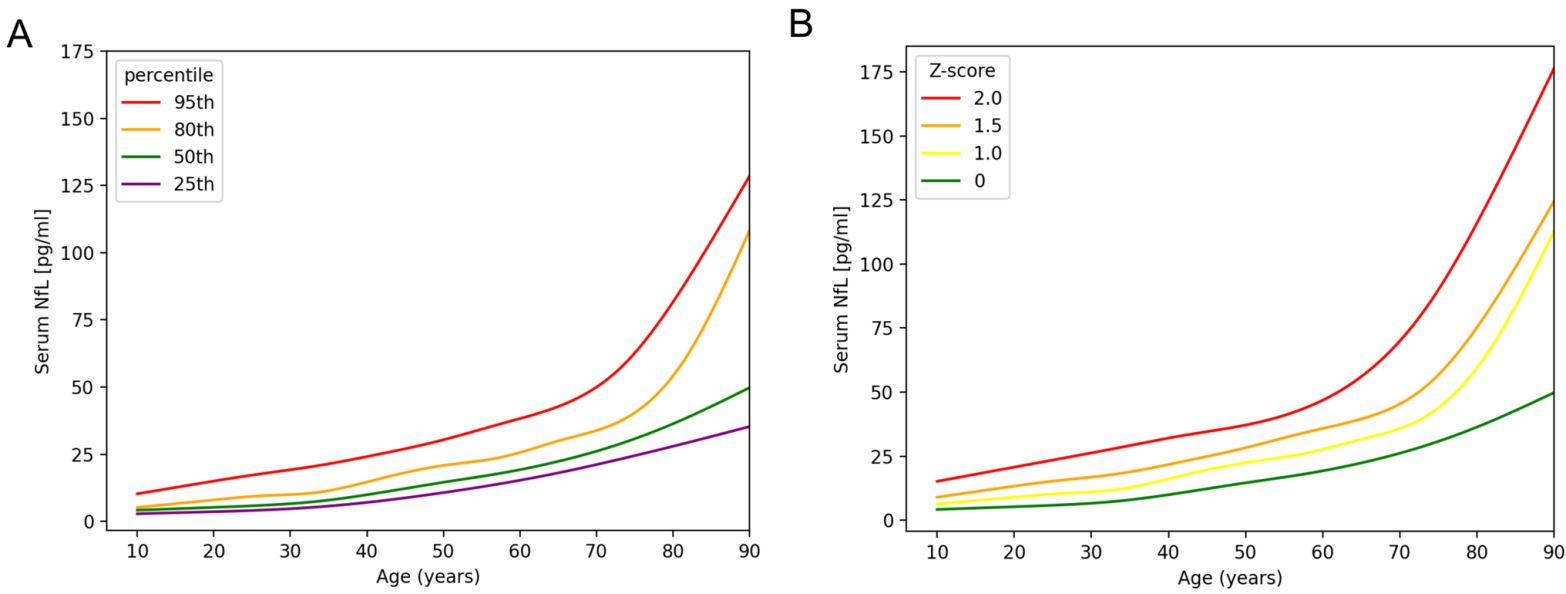
Serum NfL age reference curves. **A** The serum NfL levels as percentiles from 25 to 95. Serum NfL concentrations strongly increase over time. **B** The serum NfL concentrations as z-scores ranging from 0 to 2. For modeling the NfL concentration with age additive quantile regression was applied. Abbreviations: *NfL* neurofilament light chain

Figure 1A depicts the individual serum NfL values for patients aged 18 to 89, as well as the calculated regression lines for the median and the 25th, 80th, and 95th percentiles. The serum NfL values demonstrate an increase with age, with a median (interquartile range) of 5.5 pg/ml (4.0–7.0 pg/ml) in the group of patients below 25 years of age and 42.5 pg/ml (29.5–63.5 pg/ml) in patients above 80 years of age. The percentage increase of serum NfL per year was determined to be 3.2%. The correlation between age and serum NfL levels was found to be strong, with a Spearman *r* of 0.81 (95% CI 0.78–0.83), *p* < 0.0001. Figure 1B displays the z-scores for ranges from 0 to 2, rather than percentiles.

**Fig. 2:**
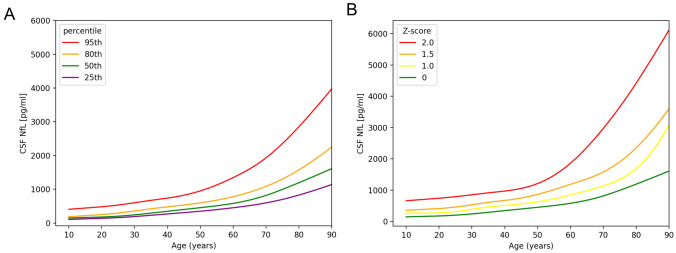
CSF NfL age reference curves. **A** The CSF NfL levels as percentiles from 25 to 95. The CSF NfL levels demonstrate a clear increase with age. **B** The CSF NfL concentrations as z-scores ranging from 0 to 2. For modeling the NfL concentration with age additive quantile regression was applied. Abbreviations: *CSF* cerebrospinal fluid, *NfL* neurofilament light chain

Figure 2 displays CSF NfL levels which increase from 172 (148–210) pg/ml in the group aged below 25 to 1322 (1084–1820) pg/ml in the age group above 80. The percentage increase of CSF NfL per year was determined to be 3.0%. Figure 2A shows the percentiles, while Fig. 2B shows the CSF NfL z-scores ranging from 0 to 2. As serum NfL levels correlate with age, CSF NfL concentrations are also associated, with an *r* of 0.82 (95% CI 0.79–0.85), *p* < 0.0001.

The serum and CSF NfL median and 95th percentiles for the respective age groups are displayed in Table 2. Z-score values are provided in the supplementary materials (Table S2).

**Table 2:** Age-specific 50% and 95% NfL percentiles in serum and CSF in control patients

Age [years]	Serum 50th percentile [pg/ml]	Serum 95th percentile [pg/ml]	CSF 50th percentile [pg/ml]	CSF 95th percentile [pg/ml]
20	5	15	175	479
25	6	17	200	530
30	7	19	240	598
35	8	21	291	669
40	10	24	344	736
45	12	27	398	821
50	15	30	452	951
55	17	34	508	1126
60	19	38	578	1343
65	22	43	678	1605
70	26	50	816	1933
75	31	63	995	2359
80	36	82	1192	2851
85	43	105	1400	3400

*CSF* Cerebrospinal fluid, *NfL* neurofilament light chain

**Fig. 3:**
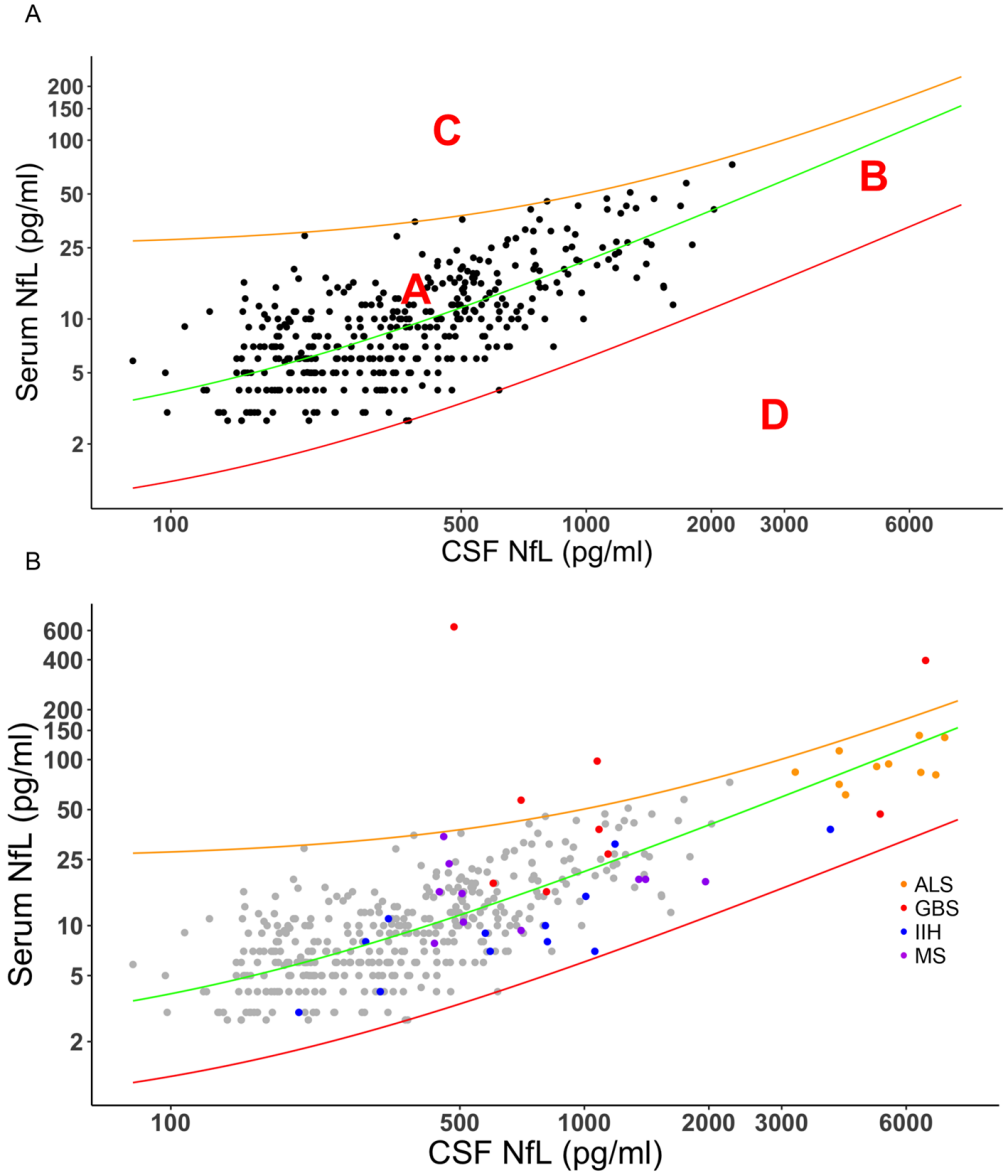
CSF versus serum NfL. Figure (**A**) displays the corresponding CSF and serum NfL values of 384 control patients. In green the median simple linear regression line is shown (R^2^ = 0.55). Orange and red lines depict the corresponding + 3SD and −3SD lines. The letters **A**–**D** define areas indicative of (**A**) normal, (**B**) increased CSF and serum, (**C**) only or predominantly increased serum and (**D**) only increased CSF NfL values. The orange and red lines represent the + 3SD and – 3SD regression lines. In (**B**) the same graph is illustrated including patients of four disease cohorts (ALS, MS, GBS, and IIH). Abbreviations: *ALS* amyotrophic lateral sclerosis, *CSF* cerebrospinal fluid, *GBS* Guillain–Barré syndrome, *IIH* Idiopathic intracranial hypertension, *NfL* neurofilament light chain, *MS* multiple sclerosis, *SD* standard deviation

A total of 384 patients had CSF and serum NfL values available for analysis in a bi-compartmental model. As illustrated in Fig. 3A, the correlation analysis between serum and CSF NfL values revealed a medium to strong correlation (*r* = 0.67 (95% CI 0.60–0.72), *p* < 0.0001). The letters A–D define different areas indicative of not elevated (A), elevated CSF and serum (B), only elevated serum (C), and only elevated CSF (D) NfL values compared to the control cohort. In a next step, we employed the bi-compartmental model from Fig. 3A in a cohort of different neurological diseases. Four patient cohorts consisting of MS, ALS, GBS and IIH were analyzed for absolute CSF and serum NfL values and z-scores (Fig. S2). The boxplots in Fig. S3 in the supplementary materials depict that the CSF to serum ratio of NfL is similar for MS and ALS but seems to be lower for GBS and higher for IIH.

In Fig. 3B, the four disease groups were illustrated in the generated graph from Fig. 3A. The MS patient group can be found in area A and the ALS group in B. GBS patients depicted high serum levels compared to CSF and can therefore partly be seen in area C. The IIH patient cohort depicted the highest CSF to serum ratio, which is consequently below the median regression line. However, no patient could be detected in area D.

The mean ratio of CSF and serum NfL in the control cohort was determined to be 44. Figure S4 in the supplements depicts the quotient of control CSF and serum NfL dependent on age. With an r of 0.01 (95%CI – 0.1–0.1, *p* = 0.9) we found no correlation.

The effect of the body mass index and sex on NfL levels is shown in the supplementary materials (Tables S3, S4, and S5, Figures S5 and S6).

## Discussion

The present study for the first time describes NfL values in serum and CSF depending on age analyzed by microfluidic Ella analysis. Our results confirm the current literature and age NfL reference values analyzed with Simoa as both serum and CSF NfL concentrations increase with age [3, 22, 29]. Moreover, our findings support the notion that the correlation between age and NfL elevation is not linear [3, 29].

We found a prominent elevation of NfL values above the age of 80 for both serum and CSF NfL. This effect was found to be quite strong and might be in addition to the normal aging effect also be attributed to a not yet detectable presymptomatic neurodegeneration in these patients. Nevertheless, this finding is significant as it underscores the necessity for caution when interpreting NfL levels in individuals beyond the age of 80.

NfL z-scores indicate the number of standard deviations a single value is above the mean of all measured control patients. In comparison to absolute values, z-scores exhibit several advantages. They are normally distributed, can also have negative values, and are independent of the platform applied to measure the NfL concentrations. Our findings display a similar blood z-score pattern as published by Benkert and colleagues [3]. Furthermore, we calculated z-scores for CSF NfL, which were not previously available in the literature.

Our correlation analysis of nearly 400 samples revealed a strong association between CSF and serum NfL values. This corroborates data from a meta-analysis on the correlation of blood and CSF NfL, which found a nearly identical *r* value [1]. However, the correlation is weaker than in our recent NfL study [12], where neurodegenerative diseases, especially ALS, were included in the correlation analysis. Nevertheless, the graphical illustration of the association of CSF and serum NfL concentrations in control patients could be beneficial for the interpretation of patient NfL values in the clinic. If CSF and blood NfL patient values are available, the clinician will be able to compare the plotted values to those of the controls as depicted in our bi-compartmental model. This would provide further insight into whether a possible elevation in blood is CNS or PNS derived, depending in which area (A–D) the value is located. Area A represents normal NfL levels. In addition to the control, in our study the MS patients are found in the upper part of this area which corroborates data of NfL in MS being slightly increased compared to healthy individuals in the same age range but only reach really high levels during an active relapse [9]. Area B depicts increased NfL levels in CSF and blood, which is highly indicative of a CNS-derived neurodegeneration. The ALS cohort as a classical CNS-derived disease group could be detected in this area confirming the hypothesis. Area C represents predominantly increased levels in blood which could be indicative of a more peripheral origin of axonal damage as seen in some neuropathies [16, 21]. Some patients of the GBS cohort could be detected in the area which is highly suspicious of a strong peripheral neuroaxonal damage for these patients. The model thereby was also able to help in the differential diagnosis to ALS. Area D, which exhibits elevated levels exclusively in CSF, is the most challenging to interpret as CSF NfL levels should also reach the bloodstream. A possible explanation could be an impaired CSF outflow as demonstrated by Engel and colleagues [7]. However, our cohort of IIH patients, which also depicted a high CSF to serum NfL ratio as seen by the colleagues [7], could also not be detected in this area. It remains to be seen if any disease group can be attributed to this area. Furthermore, it should be noted that the bi-compartmental graph is a preliminary model and in order to clearly define the areas A–D, a much larger sample size is required than was available for this study.

The average CSF NfL levels were found to be 44 times higher than those in blood, consistent with Simoa results, which detected levels approximately 42 times higher in CSF than in blood [6]. In contrast to CSF or serum NfL values alone, the ratio of both does not appear to be associated with age, indicating that the outflow of NfL from the CSF into the bloodstream may be independent of age.

The present study contributes to the existing literature as it is highly important to validate NfL data so far mostly only generated with the Simoa assay, also using other platforms. In addition, it is crucial for Ella NfL users to compare their NfL concentrations to reference values generated with the same platform as the absolute values of Simoa and Ella are not interchangeable [11, 19, 25] and harmonization projects with NfL reference materials are under way but are far from being implemented [2].

It should be noted that the present study is not without limitations. Firstly, the CSF control cohort is smaller than the serum cohort. Secondly, in the age group above 80, there is a need for further analysis of a larger number of patients.

## Conclusions

In conclusion, the age-specific reference NfL percentiles for serum and CSF can be applied to compare analyzed patient data in the clinic. Furthermore, NfL z-scores could be especially valuable in the follow-up measurements of patients, for instance under treatment. Additionally, the simultaneous measurement of NfL in CSF and blood might assist in the identification of the origin of axonal damage and help in the differential diagnosis of neurological diseases.

## Supplementary Information

Below is the link to the electronic supplementary material.

Supplementary file1 (DOCX 888 KB)

## Data Availability

All data produced in the present study are available upon reasonable request to the corresponding author.

## References

[1] Alagaratnam J, von Widekind S, De Francesco D, Underwood J, Edison P, Winston A, Zetterberg H, Fidler S (2021) Correlation between CSF and blood neurofilament light chain protein: a systematic review and meta-analysis. BMJ neurology open 3:e000143. 10.1136/bmjno-2021-000143 34223154 10.1136/bmjno-2021-000143 PMC8211066

[2] Andreasson U, Gobom J, Delatour V, Auclair G, Noam Y, Lee S, Wen J, Jeromin A, Arslan B, Maceski A, Willemse E, Zetterberg H, Kuhle J, Blennow K (2023) Assessing the commutability of candidate reference materials for the harmonization of neurofilament light measurements in blood. Clin Chem Lab Med 61:1245–1254. 10.1515/cclm-2022-1181 36709509 10.1515/cclm-2022-1181

[3] Benkert P, Meier S, Schaedelin S, Manouchehrinia A, Yaldizli O, Maceski A, Oechtering J, Achtnichts L, Conen D, Derfuss T, Lalive PH, Mueller C, Muller S, Naegelin Y, Oksenberg JR, Pot C, Salmen A, Willemse E, Kockum I, Blennow K, Zetterberg H, Gobbi C, Kappos L, Wiendl H, Berger K, Sormani MP, Granziera C, Piehl F, Leppert D, Kuhle J (2022) Serum neurofilament light chain for individual prognostication of disease activity in people with multiple sclerosis: a retrospective modelling and validation study. Lancet Neurol 21:246–257. 10.1016/S1474-4422(22)00009-6 35182510 10.1016/S1474-4422(22)00009-6

[4] Bittner S, Oh J, Havrdova EK, Tintore M, Zipp F (2021) The potential of serum neurofilament as biomarker for multiple sclerosis. Brain 144:2954–2963. 10.1093/brain/awab241 34180982 10.1093/brain/awab241 PMC8634125

[5] Brooks BR, Miller RG, Swash M, Munsat TL (2000) El escorial revisited: revised criteria for the diagnosis of amyotrophic lateral sclerosis. Amyotroph Lateral Scler Other Motor Neuron Disord 1:293–299. 10.1080/146608200300079536 11464847 10.1080/146608200300079536

[6] Disanto G, Barro C, Benkert P, Naegelin Y, Schadelin S, Giardiello A, Zecca C, Blennow K, Zetterberg H, Leppert D, Kappos L, Gobbi C, Kuhle J (2017) Serum neurofilament light: a biomarker of neuronal damage in multiple sclerosis. Ann Neurol 81:857–870. 10.1002/ana.24954 28512753 10.1002/ana.24954 PMC5519945

[7] Engel S, Halcour J, Ellwardt E, Uphaus T, Steffen F, Zipp F, Bittner S, Luessi F (2023) Elevated neurofilament light chain CSF/serum ratio indicates impaired CSF outflow in idiopathic intracranial hypertension. Fluids Barriers CNS 20:3. 10.1186/s12987-022-00403-2 36631830 10.1186/s12987-022-00403-2 PMC9832777

[8] Fitzgerald KC, Sotirchos ES, Smith MD, Lord HN, DuVal A, Mowry EM, Calabresi PA (2022) Contributors to serum NfL levels in people without neurologic disease. Ann Neurol 92:688–698. 10.1002/ana.26446 35730070 10.1002/ana.26446 PMC9489658

[9] Freedman MS, Gnanapavan S, Booth RA, Calabresi PA, Khalil M, Kuhle J, Lycke J, Olsson T (2024) Guidance for use of neurofilament light chain as a cerebrospinal fluid and blood biomarker in multiple sclerosis management. EBioMedicine 101:104970. 10.1016/j.ebiom.2024.104970 38354532 10.1016/j.ebiom.2024.104970 PMC10875256

[10] Gaetani L, Blennow K, Calabresi P, Di Filippo M, Parnetti L, Zetterberg H (2019) Neurofilament light chain as a biomarker in neurological disorders. J Neurol Neurosurg Psychiatry 90:870–881. 10.1136/jnnp-2018-320106 30967444 10.1136/jnnp-2018-320106

[11] Gauthier A, Viel S, Perret M, Brocard G, Casey R, Lombard C, Laurent-Chabalier S, Debouverie M, Edan G, Vukusic S, Lebrun-Frenay C, De Seze J, Laplaud DA, Castelnovo G, Gout O, Ruet A, Moreau T, Casez O, Clavelou P, Berger E, Zephir H, Trouillet-Assant S, Thouvenot E (2021) Comparison of Simoa(TM) and Ella(TM) to assess serum neurofilament-light chain in multiple sclerosis. Ann Clin Transl Neurol 8:1141–1150. 10.1002/acn3.51355 33830650 10.1002/acn3.51355 PMC8108418

[12] Halbgebauer S, Steinacker P, Verde F, Weishaupt J, Oeckl P, von Arnim C, Dorst J, Feneberg E, Mayer B, Rosenbohm A, Silani V, Ludolph AC, Otto M (2022) Comparison of CSF and serum neurofilament light and heavy chain as differential diagnostic biomarkers for ALS. J Neurol Neurosurg Psychiatry 93:68–74. 10.1136/jnnp-2021-327129 34417339 10.1136/jnnp-2021-327129

[13] Karantali E, Kazis D, Chatzikonstantinou S, Petridis F, Mavroudis I (2021) The role of neurofilament light chain in frontotemporal dementia: a meta-analysis. Aging Clin Exp Res 33:869–881. 10.1007/s40520-020-01554-8 32306372 10.1007/s40520-020-01554-8

[14] Koini M, Pirpamer L, Hofer E, Buchmann A, Pinter D, Ropele S, Enzinger C, Benkert P, Leppert D, Kuhle J, Schmidt R, Khalil M (2021) Factors influencing serum neurofilament light chain levels in normal aging. Aging 13:25729–25738 10.18632/aging.203790 10.18632/aging.203790 PMC8751593 34923481

[15] Leonhard SE, Mandarakas MR, Gondim FAA, Bateman K, Ferreira MLB, Cornblath DR, van Doorn PA, Dourado ME, Hughes RAC, Islam B, Kusunoki S, Pardo CA, Reisin R, Sejvar JJ, Shahrizaila N, Soares C, Umapathi T, Wang Y, Yiu EM, Willison HJ, Jacobs BC (2019) Diagnosis and management of Guillain–Barré syndrome in ten steps. Nat Rev Neurol 15:671–683. 10.1038/s41582-019-0250-9 31541214 10.1038/s41582-019-0250-9 PMC6821638

[16] Mariotto S, Farinazzo A, Magliozzi R, Alberti D, Monaco S, Ferrari S (2018) Serum and cerebrospinal neurofilament light chain levels in patients with acquired peripheral neuropathies. JPNS 23:174–177. 10.1111/jns.12279 29974556 10.1111/jns.12279

[17] Mollan SP, Davies B, Silver NC, Shaw S, Mallucci CL, Wakerley BR, Krishnan A, Chavda SV, Ramalingam S, Edwards J, Hemmings K, Williamson M, Burdon MA, Hassan-Smith G, Digre K, Liu GT, Jensen RH, Sinclair AJ (2018) Idiopathic intracranial hypertension: consensus guidelines on management. J Neurol Neurosurg Psychiatry 89:1088–1100. 10.1136/jnnp-2017-317440 29903905 10.1136/jnnp-2017-317440 PMC6166610

[18] Nagel G, Unal H, Rosenbohm A, Ludolph AC, Rothenbacher D (2013) Implementation of a population-based epidemiological rare disease registry: study protocol of the amyotrophic lateral sclerosis (ALS)–registry Swabia. BMC Neurol 13:22. 10.1186/1471-2377-13-22 23414001 10.1186/1471-2377-13-22 PMC3582473

[19] Notzel M, Werder LI, Ziemssen T, Akgun K (2022) Ella versus Simoa serum neurofilament assessment to monitor treatment response in highly active multiple sclerosis patients. Int J Mol Sci. 10.3390/ijms232012361 36293227 10.3390/ijms232012361 PMC9604350

[20] Rosenbohm A, Peter RS, Erhardt S, Lule D, Rothenbacher D, Ludolph AC, Nagel G (2017) Epidemiology of amyotrophic lateral sclerosis in southern Germany. J Neurol 264:749–757. 10.1007/s00415-017-8413-3 28220290 10.1007/s00415-017-8413-3

[21] Sandelius A, Zetterberg H, Blennow K, Adiutori R, Malaspina A, Laura M, Reilly MM, Rossor AM (2018) Plasma neurofilament light chain concentration in the inherited peripheral neuropathies. Neurology 90:e518–e524. 10.1212/WNL.0000000000004932 29321234 10.1212/WNL.0000000000004932 PMC5818017

[22] Simren J, Andreasson U, Gobom J, Suarez Calvet M, Borroni B, Gillberg C, Nyberg L, Ghidoni R, Fernell E, Johnson M, Depypere H, Hansson C, Jonsdottir IH, Zetterberg H, Blennow K (2022) Establishment of reference values for plasma neurofilament light based on healthy individuals aged 5–90 years. Brain Commun 4:fcac174. 10.1093/braincomms/fcac174 35865350 10.1093/braincomms/fcac174 PMC9297091

[23] Steinacker P, Feneberg E, Weishaupt J, Brettschneider J, Tumani H, Andersen PM, von Arnim CA, Bohm S, Kassubek J, Kubisch C, Lule D, Muller HP, Muche R, Pinkhardt E, Oeckl P, Rosenbohm A, Anderl-Straub S, Volk AE, Weydt P, Ludolph AC, Otto M (2016) Neurofilaments in the diagnosis of motoneuron diseases: a prospective study on 455 patients. J Neurol Neurosurg Psychiatry 87:12–20. 10.1136/jnnp-2015-311387 26296871 10.1136/jnnp-2015-311387

[24] Thompson AJ, Banwell BL, Barkhof F, Carroll WM, Coetzee T, Comi G, Correale J, Fazekas F, Filippi M, Freedman MS, Fujihara K, Galetta SL, Hartung HP, Kappos L, Lublin FD, Marrie RA, Miller AE, Miller DH, Montalban X, Mowry EM, Sorensen PS, Tintore M, Traboulsee AL, Trojano M, Uitdehaag BMJ, Vukusic S, Waubant E, Weinshenker BG, Reingold SC, Cohen JA (2018) Diagnosis of multiple sclerosis: 2017 revisions of the McDonald criteria. Lancet Neurol 17:162–173. 10.1016/S1474-4422(17)30470-2 29275977 10.1016/S1474-4422(17)30470-2

[25] Truffi M, Garofalo M, Ricciardi A, Cotta Ramusino M, Perini G, Scaranzin S, Gastaldi M, Albasini S, Costa A, Chiavetta V, Corsi F, Morasso C, Gagliardi S (2023) Neurofilament-light chain quantification by Simoa and Ella in plasma from patients with dementia: a comparative study. Sci Rep 13:4041. 10.1038/s41598-023-29704-8 36899015 10.1038/s41598-023-29704-8 PMC10006166

[26] Uenal H, Rosenbohm A, Kufeldt J, Weydt P, Goder K, Ludolph A, Rothenbacher D, Nagel G (2014) Incidence and geographical variation of amyotrophic lateral sclerosis (ALS) in southern Germany–completeness of the ALS registry Swabia. PLoS ONE 9:e93932. 10.1371/journal.pone.0093932 24722455 10.1371/journal.pone.0093932 PMC3983245

[27] Verde F, Otto M, Silani V (2021) Neurofilament light chain as biomarker for amyotrophic lateral sclerosis and frontotemporal dementia. Front Neurosci 15:679199. 10.3389/fnins.2021.679199 34234641 10.3389/fnins.2021.679199 PMC8255624

[28] Verde F, Steinacker P, Weishaupt JH, Kassubek J, Oeckl P, Halbgebauer S, Tumani H, von Arnim CAF, Dorst J, Feneberg E, Mayer B, Muller HP, Gorges M, Rosenbohm A, Volk AE, Silani V, Ludolph AC, Otto M (2019) Neurofilament light chain in serum for the diagnosis of amyotrophic lateral sclerosis. J Neurol Neurosurg Psychiatry 90:157–164. 10.1136/jnnp-2018-318704 30309882 10.1136/jnnp-2018-318704

[29] Vermunt L, Otte M, Verberk IMW, Killestein J, Lemstra AW, van der Flier WM, Pijnenburg YAL, Vijverberg EGB, Bouwman FH, Gravesteijn G, van de Berg WDJ, Scheltens P, van Harten AC, Willemse EAJ, Teunissen CE (2022) Age- and disease-specific reference values for neurofilament light presented in an online interactive support interface. Ann Clin Transl Neurol 9:1832–1837. 10.1002/acn3.51676 36196979 10.1002/acn3.51676 PMC9639622

[30] Witzel S, Huss A, Nagel G, Rosenbohm A, Rothenbacher D, Peter RS, Bazner H, Bortlein A, Dempewolf S, Schabet M, Hecht M, Kohler A, Opherk C, Naegele A, Sommer N, Lindner A, Alexudis C, Bachhuber F, Halbgebauer S, Brenner D, Ruf W, Weiland U, Mayer B, Schuster J, Dorst J, Tumani H, Ludolph AC (2024) Population-based evidence for the use of serum neurofilaments as individual diagnostic and prognostic biomarkers in amyotrophic lateral sclerosis. Ann Neurol. 10.1002/ana.27054 39177232 10.1002/ana.27054

[31] Yuan A, Rao MV, Veeranna Nixon RA (2017) Neurofilaments and Neurofilament Proteins in Health and Disease. Cold Spring Harbor Perspect Biol. 10.1101/cshperspect.a018309 10.1101/cshperspect.a018309 PMC5378049 28373358

[32] Yuan A, Rao MV, Veeranna NRA (2012) Neurofilaments at a glance. J Cell Sci 125:3257–3263. 10.1242/jcs.104729 22956720 10.1242/jcs.104729 PMC3516374

